# Enhanced Recovery of Food-Grade *Euglena gracilis* Biomass Through Synergistic pH-Modified Chitosan Flocculation and Green Light Stimulation

**DOI:** 10.3390/microorganisms13020303

**Published:** 2025-01-30

**Authors:** Jiangyu Zhu, Lan Yang, Li Ding, Zhengfei Yang, Yongqi Yin, Minato Wakisaka, Shahram Ashouri, Mohammadhadi Jazini, Weiming Fang

**Affiliations:** 1 School of Food Science and Engineering, Yangzhou University, Yangzhou 225127, China; 008051@yzu.edu.cn (J.Z.); yzf@yzu.edu.cn (Z.Y.); yqyin@yzu.edu.cn (Y.Y.); 2 School of Textile Science and Engineering, Tiangong University, Tianjin 300387, China; 3 Food Study Centre, Fukuoka Women’s University, 1-1-1 Kasumigaoka, Fukuoka 813-8529, Japan; wakisaka@fwu.ac.jp; 4 Department of Chemical Engineering, Isfahan University of Technology, Isfahan 84156-83111, Iran; s.ashouri@ce.iut.ac.ir (S.A.); m.h.jazini@iut.ac.ir (M.J.)

**Keywords:** *Euglena gracilis*, harvesting, response surface methodology, chitosan, chlorophyll fluorescence

## Abstract

The efficient and cost-effective harvesting of food-grade *Euglena gracilis* remains a critical challenge in microalgal food production. This study presents an innovative, food-safe approach integrating pH preconditioning, chitosan biopolymer flocculation, and green light irradiation to leverage *E. gracilis’* natural phototactic behavior. Response surface methodology optimized the parameters (pH 6.49, 46.10 mg·L^−1^ chitosan, and 60 min green light), achieving 93.07% biomass recovery, closely matching the predicted 92.21%. The synergistic effects of pH-modified chitosan flocculation and phototaxis significantly enhanced the harvesting efficiency compared to conventional methods. Notably, harvested cells maintained substantial photosynthetic capability, as evidenced by chlorophyll fluorescence analysis, ensuring the preservation of nutritional quality. Economic analysis revealed exceptional harvesting cost-effectiveness at 2.35 USD per kg of dry weight biomass harvested. The method’s use of food-grade chitosan and non-invasive light stimulation ensures product safety while minimizing the environmental impact. This sustainable and economical approach offers a promising solution for industrial-scale production of food-grade *E. gracilis* while demonstrating potential applicability to other phototactic microalgae species.

## 1. Introduction

Microalgae have gained significant attention as a sustainable source of biomass for various applications, including biofuels, nutraceuticals, and environmental remediation [1]. However, the low biomass concentration in microalgal cultures and the small cell size of microalgae pose significant challenges for efficient and cost-effective harvesting processes [2]. Conventional harvesting methods, such as centrifugation and filtration, are often energy-intensive and economically unfeasible for large-scale operations, necessitating the exploration of alternative approaches. Centrifugation, a widely used harvesting technique, incurs significant operational costs. Studies have reported that the energy consumption ranges from 0.8 to 9 kWh·m^−3^ for microalgal culture [3]. Additionally, Brentner et al. [4] highlighted that centrifugation energy costs can constitute up to 30–40% of total microalgal biomass production expenses, making it economically challenging for large-scale operations.

Among various microalgae species, *Euglena gracilis* has emerged as a promising candidate due to its unique characteristics and versatile applications. Unlike many other microalgae, *E. gracilis* lacks a rigid cell wall, instead possessing a flexible pellicle [5]. This distinctive feature makes it an attractive option for the production of high-value compounds such as paramylon, α-tocopherol, and other nutraceuticals [5]. Additionally, *E. gracilis* has shown potential in biofuel production and environmental remediation [6]. However, the absence of a rigid cell wall presents unique challenges in harvesting as conventional methods optimized for other microalgae species may not be directly applicable or efficient for *E. gracilis*.

Flocculation has been widely studied as a cost-effective harvesting method for microalgae [2]. While inorganic flocculants are effective, they often contaminate the biomass and pose environmental concerns [7]. In contrast, biopolymer flocculants like chitosan offer a more sustainable alternative. Chitosan, derived from chitin, is biodegradable, non-toxic, and effective at low dosages [8]. However, the application of chitosan for *E. gracilis* harvesting remains underexplored, and its effectiveness may be influenced by factors such as pH and the unique cellular structure of this microalga. Another distinctive characteristic of *E. gracilis* that can be leveraged for harvesting is its phototactic behavior. Phototaxis, the movement of organisms in response to light, is particularly pronounced in *E. gracilis*, which exhibits positive phototaxis toward certain wavelengths (500–560 nm), especially in the green spectrum [9]. While this behavior has been studied extensively in *E. gracilis* biology, its potential application in harvesting processes has not been fully explored.

This study presents a novel, synergistic approach to *E. gracilis* harvesting by integrating three key elements: pH preconditioning, chitosan flocculation, and green light irradiation. By combining these techniques, we aim to leverage both the surface charge properties and the unique phototactic behavior of *E. gracilis* to enhance harvesting efficiency. This approach not only addresses the challenges posed by the lack of a rigid cell wall in *E. gracilis* but also offers a potential template for harvesting other phototactic microalgae species. Response surface methodology (RSM) was employed to optimize the key parameters of this integrated process, namely chitosan concentration, light exposure duration, and pH. Furthermore, we assessed the viability of harvested cells through chlorophyll fluorescence analysis, addressing a critical concern in microalgae harvesting—the maintenance of cell integrity and functionality post-harvest.

By synergistically combining pH preconditioning, chitosan flocculation, and phototaxis, this study aims to overcome the limitations of conventional harvesting methods for *E. gracilis*, offering a more efficient and sustainable approach to biomass recovery for this economically important microalga. The findings of this research contribute to the development of cost-effective and environmentally friendly harvesting techniques, potentially advancing the commercial viability of *E. gracilis* cultivation and its diverse applications.

## 2. Materials and Methods

### 2.1. Microalgae Cultivation Conditions

The *Euglena gracilis* Klebs (FACHB-850; Klebs, 1883) strain was obtained from the Freshwater Algae Culture Collection at the Institute of Hydrobiology, Chinese Academy of Sciences (Wuhan, China). HUT medium was prepared according to Hutner et al. [10] and sterilized at 121 °C for 20 min under 1.05 kg/cm^2^ pressure before use. The appropriate amount of algal stock solution was inoculated into the medium at the ratio of 1:9. The culture was maintained in 250 mL Erlenmeyer flasks with a culture volume of 100 mL, on a horizontal orbital shaker (IKA KS 260 basic, Königswinter, Germany) set at 120 rpm to ensure uniform mixing and prevent algal attachment or sedimentation. The culture temperature was maintained at 25 ± 1 °C with a light intensity of 5000 lx and a 12-h-light–12-h-dark photoperiod. No additional gas was supplemented during the cultivation, and each flask was fitted with a breathable rubber plug. The culture was used during the logarithmic growth phase. At the start of the harvesting experiment, the dry weight (DW, mg·L^−1^) of the algal solution was determined using a previously determined conversion formula:

DW = 1007.5 × OD_680_ + 2.7454 (R^2^ = 0.9997) (1)

To establish this conversion formula relating optical density at 680 nm (OD680) to dry weight concentration, we followed a standard gravimetric method. Briefly, *E. gracilis* cultures were harvested by vacuum filtration using pre-weighed, oven-dried (105 °C, 24 h) glass fiber filters (Whatman GF/C, pore size 0.45 μm). After filtration, the filters with biomass were carefully washed with deionized water to remove salt residues, then dried again at 105 °C for 24 h and weighed. The dry weight was calculated by the difference in filter weight before and after filtration. Multiple samples across different cell densities were used to develop the linear regression model correlating optical density at 680 nm with dry weight concentration.

### 2.2. Harvesting Experiment with Chitosan, pH Pretreatment, and Green Light Irradiation

To prepare the chitosan stock solution with a mass concentration of 5 g·L^−1^, 250 mg of chitosan (deacetylation degree of chitosan ≥ 90%) was added into 50 mL of 1% (*v*/*v*) acetic acid solution and stirred with a magnetic stirrer for 20 min. Subsequently, the stock solution was diluted into the algal solution to obtain varying concentrations of chitosan addition. The initial pH of the algal solution was measured using a pH meter (PHS-2F, Shanghai Yidian Technology Co., Ltd., Shanghai, China), and pH pretreatment was achieved by adjusting with 1 M NaOH or HCl solution.

**Figure 1:**
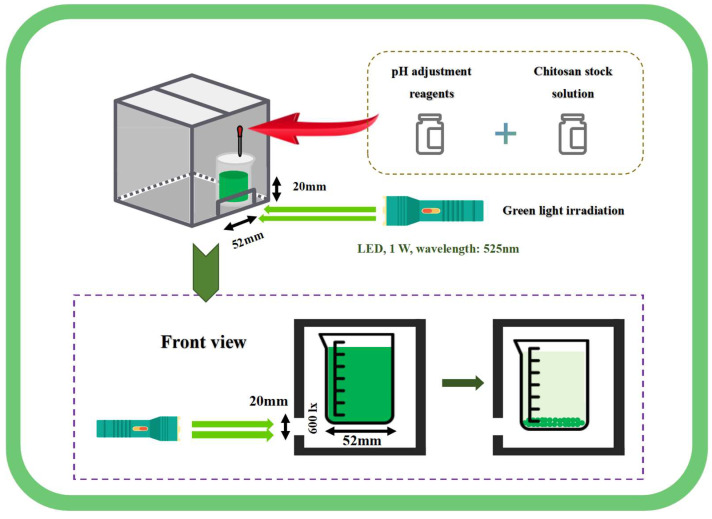
Schematic diagram of harvest by green light irradiation combined with pH pretreatment and chitosan flocculation.

The green light, with a wavelength of 525 nm, was provided by a 1 W LED light source (E17, AloneFire, Shenzhen, China), and projected in a dark chamber. The schematic diagram is shown in Figure 1. A small opening measuring 52 mm ×20 mm was cut below the sealed dark chamber, with 100 mL of algal solution placed in a 100 mL beaker (beaker diameter: 52 mm). The beaker was placed in the dark chamber, adjacent to the small hole. The green light was projected in a straight line through the small opening to prevent refraction. The light intensity on the algal solution below was 600 lx. The initial density of algal culture in all treatment groups was 0.53 g·L^−1^. Regardless of whether the green light treatment was active or the duration of exposure, the static harvesting time was set at 60 min. The algal solution was left to settle in the dark chamber, except during the green light exposure period.

### 2.3. Determination of the Range of pH, Chitosan Concentration, and Green Light Irradiation Time

*E. gracilis* was pre-cultured in HUT medium until reaching the exponential growth phase. For experimental cultures, 10 mL of the pre-culture was inoculated into 90 mL of HUT medium (control) in 250 mL Erlenmeyer flasks. Cultures were incubated at 20 ± 1 °C under 5000 lx illumination with a 12-h–12-h light–dark cycle in a controlled environment chamber. Flasks were manually shaken 5–6 times daily to prevent cell adhesion and ensure uniform light exposure.

The selected pH range was informed by the protonation behavior of chitosan. At lower pH values, the amine groups (-NH_2_) on chitosan become protonated (-NH_3_^+^), increasing the polymer’s positive charge density. This protonation is crucial for effective flocculation as the positively charged chitosan can more readily interact with negatively charged microalgal cell surfaces through electrostatic interactions. Preliminary tests across the pH spectrum from 3 to 9 were conducted to empirically determine the optimal pH range that balances chitosan protonation, electrostatic interactions, and *E. gracilis* cell stability.

To determine the appropriate ranges for the independent variables of chitosan concentration, pH, and green light irradiation time, preliminary single-factor experiments were conducted. These aided in narrowing down the factor levels for the subsequent response surface methodology optimization experiments. Five groups of 100 mL algal solutions were taken from the culture medium, and the pH was adjusted to 3, 5, 7, and 9 by adding 1 M NaOH or HCl solution. The chitosan stock solution was added to achieve a concentration of 50 mg·L^−1^. The solutions were exposed to green light irradiation in the dark chamber and allowed to stand for 60 min. After the flocculation process, samples of the supernatant were taken 2 cm below the liquid surface at the same scale line to determine the biomass recovery (BR). The BR was calculated by measuring the optical density of the *E. gracilis* culture at 680 nm using a UV-7504C ultraviolet–visible spectrophotometer. The formula for calculating the BR is outlined below:

BR = (OD_i_ − OD_f_) ÷ OD_i_ × 100 (2)

where OD_i_ is the initial optical density; and OD_f_ is the optical density of the supernatant at the treatment time. This experiment was used to determine the range of pH values.

Correspondingly, five groups of 100 mL algal solutions were taken from the culture stock, and the chitosan stock solutions were added to achieve chitosan concentrations of 10, 50, 90, 130, and 170 mg·L^−1^. After stirring thoroughly, the samples were subjected to green light irradiation in a dark chamber and left undisturbed for 60 min. Subsequently, samples were taken 2 cm below the liquid surface at the same scale line to determine the BR. This experiment was used to determine the range of chitosan concentrations.

To determine the range of green light irradiation times, five groups of 100 mL algal solutions were taken from the culture stock, and the chitosan stock solution was added to achieve a chitosan concentration of 50 mg·L^−1^. After stirring thoroughly, the solutions were placed in the dark chamber and irradiated with green light for 10, 20, 35, 45, and 60 min (for samples with green light irradiation times less than 60 min, dark treatment was continued after the green light irradiation until the total treatment time reached 60 min). After the flocculation process, samples of the supernatant were taken 2 cm below the liquid surface at the same scale line to determine the BR.

### 2.4. Response Surface Methodology (RSM) for Optimization

Response surface methodology (RSM) with a Box–Behnken design (BBD) was employed to optimize the synergistic harvesting process by investigating the effects and interactions of three critical factors: pH (A), chitosan dosage (B), and green light irradiation time (C). The ranges for each factor were determined based on the results of preliminary single-factor experiments and practical considerations. The chosen levels for each factor in the RSM design were as follows: A: pH (5, 7, 9); B: chitosan dosage (20, 50, 80 mg·L^−1^); C: green light irradiation time (10, 35, 60 min).

A total of 17 experimental runs were conducted according to the BBD matrix to evaluate the combined effects of the three factors on the response variable, BR (Y). The experimental data were fitted to various regression models (linear, two-factor interaction, quadratic, and cubic), and the model exhibiting the highest R^2^ value and statistical significance was selected as the final predictive model. Central point replicates (runs 13–17) were included to evaluate experimental reproducibility and estimate the pure error. Minor variations in responses at identical factor levels reflect inherent biological variability and measurement precision, which are statistically accounted for in the model. The adequacy and significance of the chosen model were evaluated using analysis of variance (ANOVA). The quality of the fit was expressed by the coefficient of determination R^2^ and the adjusted R^2^ statistic. The statistical significance of the model terms was determined using F-tests and their associated *p*-values. Three-dimensional response surface plots were constructed to visualize the relationship between the response (biomass recovery efficiency) and any two factors while holding the third factor constant at its center point level.

The optimum levels of the three factors (pH, chitosan dosage, and green light time) that maximized BR were determined by solving the regression equation and analyzing the response surface plots. Additional confirmation experiments were conducted using the optimum conditions to validate the predicted responses from the model. The experimental values were compared with the predicted values to confirm the adequacy of the optimized model for the synergistic harvesting process.

### 2.5. Chlorophyll Fluorescence Parameters of Algal Cells Before and After Treatment

To assess the impact of the optimized harvesting treatment on the photosynthetic capability of *E. gracilis*, the chlorophyll fluorescence parameters were measured using an FP110 fluorometer (Photon Systems Instruments, Drásov, Czech Republic). The minimum fluorescence yield (F0) and the maximum fluorescence yield (Fm) were determined for both untreated and harvested *E. gracilis* cells (pH 6.49; chitosan concentration of 46.10 mg·L^−1^; green light irradiation 60 min) after a 30-min dark adaptation period, with three parallel groups. Fv/Fm, which is calculated by (Fm − F0)/Fm, represents the maximal quantum yield of photosystem II (PSII) and serves as an indicator of the light energy conversion efficiency of PSII. Another index, Fv/F0, estimates the potential photosynthetic activity as (Fm − F0)/F0 [11]. These two indices, Fv/Fm and Fv/F0, are calculated to measure photosynthetic efficiency.

### 2.6. Unit Cost of Harvesting E. gracilis

The consumption of chitosan, pH adjustment reagents, and electricity for green light irradiation was estimated according to the modified Elcik’s method [12], and the unit cost (UC; USD·kg^−1^) for harvesting *E. gracilis* biomass was calculated based on the following equation:

UC (USD·kg^−1^) = (C_ct_ + C_pH_ + C_e_) ÷ (BM × BR) × 1000 (3)

where C_ct_ is the cost of chitosan consumption (USD·L^−1^); C_pH_ is the cost of pH adjustment reagents (USD·L^−1^); C_e_ is the cost of electricity for green light irradiation (USD·L^−1^); BM is the initial dry biomass (g·L^−1^); and BR is the biomass recovery (%).

### 2.7. Statistical Analysis

The experiment was conducted in triplicate, and the data were processed using Design-Expert 13 and SPSS 17.0 software. Analysis of variance (ANOVA) was performed to assess the significance of the model terms and lack of fit. The coefficient of determination (R^2^) and adjusted R^2^ were used to evaluate the goodness of fit of the regression model. The statistical analyses included both independent-samples and paired t-tests. Specifically, paired t-tests were conducted to compare predicted and experimental values, ensuring a comprehensive statistical validation of the experimental results. Additionally, an independent-samples Student’s *t*-test was used to assess the significance of differences in chlorophyll fluorescence parameters before and after the optimized harvesting treatment. Differences were considered statistically significant at *p* < 0.05.

## 3. Results and Discussion

### 3.1. Evaluation of Operating Parameters

This study investigated a method for harvesting *E. gracilis* biomass by combining pH pretreatment with a chitosan biopolymer composite and green light irradiation process, optimizing the chitosan concentration, green light irradiation time, and pH value. Based on the BBD, RSM was employed to investigate the relative importance of the variables and the response. Table 1 shows the BR for the 17 experimental runs. The BBD runs revealed that the thirteenth run had the highest BBR of 92.22%, while the second run had the lowest efficiency of 49.85%.

The comparative data in Table 1 indicate that the BBR was mainly influenced by the variations in pH and green light irradiation time. At pH 7, the BBR was higher than 78.10%, and as the green light irradiation time increased, the BBR also increased. These observations are consistent with previous studies on the effects of pH and light on microalgae harvesting. Elcik et al. [12] reported that the mechanism of chitosan-induced interaction with microalgal cells depends on the pH value. Due to its amino groups, chitosan carries a positive charge at lower pH values, leading to electrostatic interactions with the negatively charged microalgal cells. This charge neutralization effect is crucial for effective flocculation and harvesting [7,8]. While chitosan facilitates flocculation via charge neutralization and bridging, the subsequent green light irradiation period allows for residual cell mobility due to phototaxis. This sequential process ensures that chitosan-induced flocculation does not fully immobilize cells before light exposure, enabling phototactic aggregation to enhance floc formation and settling efficiency. The observed decrease in BBR at pH 9 can be attributed to the reduced electrostatic interactions between the partially deprotonated chitosan and the algal cells. The influence of green light irradiation time on the BBR can be explained by the phototactic behavior of *E. gracilis*. Under green light exposure, *E. gracilis* cells exhibit positive phototaxis, resulting in cell aggregation and enhanced flocculation [9]. The synergistic effect of phototaxis and chitosan flocculation contributes to the improved BBR observed at longer light irradiation times. Interestingly, the influence of chitosan concentration on the flocculation performance was relatively limited compared to pH and light irradiation time. This observation aligns with the ANOVA results, where chitosan concentration had an insignificant linear effect on the BR. While an optimal chitosan dosage is necessary for effective flocculation, excessive concentrations can lead to particle restabilization and inhibit floc formation [8].

The results indicate that pH and green light irradiation time played a crucial role in the flocculation efficiency, with mildly acidic pH and green light irradiation conditions favoring more efficient flocculation of *E. gracilis*. The synergistic effects of charge neutralization by pH adjustment, chitosan flocculation, and phototaxis-induced cell aggregation contributed to the enhanced BBR observed in this study.

### 3.2. Exploration of the Effects of Three Factors on BR Using Response Surface Methodology

#### 3.2.1. Evaluation of the RSM Models

Model selection was conducted through a comprehensive statistical analysis of various regression models, including linear, two-factor interaction (2FI), quadratic, and cubic approaches. The quadratic model emerged as the most appropriate, demonstrating exceptional predictive capabilities.

The quadratic model exhibited an outstanding coefficient of determination (R^2^) of 0.9817, indicating an excellent fit to the experimental data. The adjusted R^2^ value of 0.9582 further confirmed the model’s reliability. The predicted R^2^ value of 0.8817 closely aligned with the adjusted R^2^, supporting the model’s predictive capabilities [13]. A *p*-value less than 0.05 signified the statistical significance of the quadratic model, while values exceeding 0.1 for linear, 2FI, and cubic models indicated their lack of significance. The model’s Adeq Precision—representing the signal-to-noise ratio—was greater than 4, indicating an adequate signal. Additionally, the coefficient of variation (CV) of 3.37% suggested high precision and experimental reliability. The BR variability among central point replicates (85.90–92.22%) underscores the importance of including multiple replicates to quantify experimental error. The insignificant lack of fit (*p* = 0.6060) confirms that the model adequately describes the system despite this variability. Consequently, the quadratic model was selected as the final predictive model due to its superior ability to capture the curvature and interactions present in the response surface [12].

#### 3.2.2. Statistical Analysis of Factors and Establishment of the Regression Equation

The analysis of variance (ANOVA) for the quadratic model is presented in Table 2. The model’s F-value of 41.74 and the associated low *p*-value (<0.0001) indicate that the model is statistically significant. Additionally, the lack-of-fit test has a *p*-value of 0.6060, which is greater than the significance level of 0.05, suggesting that the lack of fit is not significant relative to the pure error. The coefficient of determination (R^2^) value of 0.9817 and the adjusted R^2^ value of 0.9582 confirm the excellent fit of the model to the experimental data. The predicted R^2^ value of 0.8817 is in reasonable agreement with the adjusted R^2^ value, further supporting the model’s adequacy. Based on the ANOVA results, the quadratic model is adequate for predicting the BR within the studied factor ranges.

The ANOVA analysis also revealed that the linear terms of pH (A) and green light irradiation time (C), the interactive terms AB and AC, and the quadratic terms A^2^ and B^2^ were significant model terms affecting the BR. The chitosan concentration (B) had an insignificant linear effect, while the interactive term BC as well as quadratic term C^2^ were also insignificant. The observation indicates that pH and green light irradiation time significantly affected the harvesting of *E. gracilis*, while the dependence on chitosan concentration was relatively low. The quadratic regression equation for the three factors of pH (A), chitosan concentration (B), and green light irradiation time (C) with the biomass recovery (BR) as the response (Y) is given as follows:

Y = 89.99 − 4.58 × A + 1.44 × B + 3.36 × C + 9.55 × AB − 4.06 × AC − 0.5 × BC − 19.39 × A^2^ − 5.71 × B^2^ − 2.17 × C^2^ (4)

#### 3.2.3. Interactive Effects of Variables on BBR

**Figure 2:**
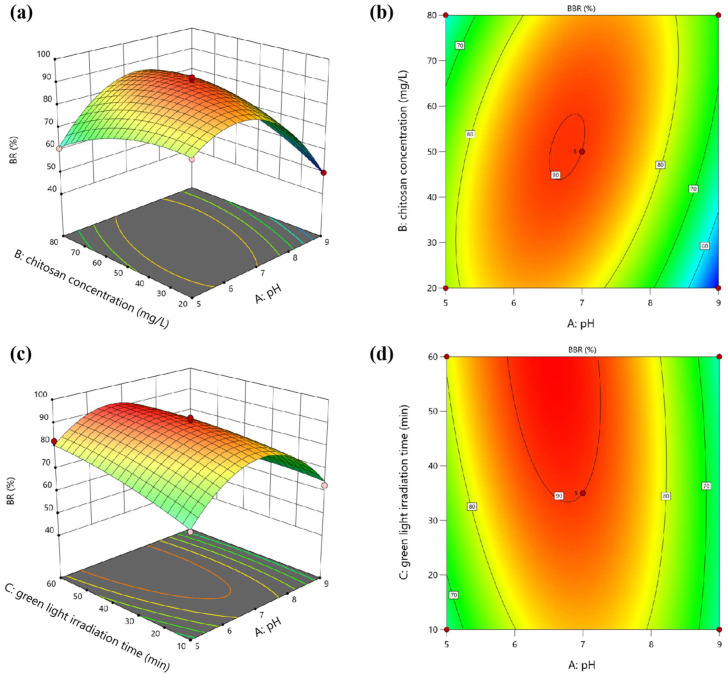
Three-dimensional response surface plots and two-dimensional contour plots showing the interactive effects of (**a**,**b**) pH and chitosan concentration, and (**c**,**d**) pH and green light irradiation time on the BR of *E. gracilis*: (**a**,**b**) interactive effects of pH and chitosan concentration on BR, with green light irradiation time held constant at 35 min; (**c**,**d**) interactive effects of pH and green light irradiation time on BR, with chitosan concentration held constant at 50 mg·L^−1^.

The interactive effects of pH, chitosan concentration, and green light irradiation time on the BR of *E. gracilis* can be visualized through three-dimensional response surface plots. Figure 2 illustrates the three-dimensional response surfaces and two-dimensional contour plots for the interactions between pairs of factors while holding the third factor constant at its center point level. The steeper the surface, the denser the contour lines, indicating a more significant influence. The more elliptical the contour lines, the stronger the interaction between the two factors [12].

The interaction between pH and chitosan concentration (green light irradiation time: 35 min), as shown in Figure 2a,b, had a significant effect on the BR as evidenced by the low *p*-value (0.0002) of the AB interactive term. The response surfaces indicate that the BR increased with an increase in pH from acidic to mildly acidic conditions, reaching a maximum at approximately pH 6.5. This trend aligns with previous findings that optimal flocculation of microalgae cells occurs at slightly acidic pH values, where the positively charged chitosan polymer can effectively interact with the negatively charged cell surfaces [14]. Additionally, the lack of a rigid cell wall in *E. gracilis* makes them more susceptible to changes in the external environment, such as pH and light conditions [15]. The acidic pH pretreatment likely facilitated the aggregation and flocculation of *E. gracilis* cells by altering the surface charge properties, enhancing the effectiveness of chitosan flocculation. However, as the pH approached neutral or alkaline conditions, the BR declined, likely due to the reduced electrostatic attraction between chitosan and algal cells [12]. The effect of chitosan concentration on the BR was relatively minor compared to pH but a synergistic effect was observed at moderate concentrations of chitosan (around 50 mg·L^−1^) and mildly acidic pH conditions. This synergy can be attributed to the improved charge neutralization and bridging effects provided by chitosan, facilitating the aggregation and flocculation of microalgal cells [7,8].

The interaction between pH and green light irradiation time was also significant (chitosan concentration: 50 mg·L^−1^), as indicated by the *p*-value (0.0167) of the AC interactive term. As shown in Figure 2c,d, prolonged green light exposure enhanced the BR, likely due to the phototactic behavior of *E. gracilis* cells, which tend to aggregate in response to green light irradiation [9]. The synergistic effect of pH pretreatment and green light irradiation was evident, with the highest BR achieved at a combination of mildly acidic pH and extended green light exposure. This synergy can be attributed to the enhanced flocculation efficiency under mildly acidic conditions, coupled with the phototactic cell aggregation induced by green light irradiation. In contrast, the interaction between chitosan concentration and green light irradiation time had an insignificant effect on the BR as indicated by the high *p*-value (0.7116) of the BC interactive term. Green light irradiation time had a more substantial impact on the BR compared to chitosan concentration. Maximum recovery was observed at higher green light exposure times (around 60 min) and moderate chitosan concentrations (50–60 mg·L^−1^). This suggests that while chitosan concentration plays a role in facilitating flocculation, the phototactic response induced by green light irradiation is the dominant factor influencing the BBR of *E. gracilis*.

Overall, the results highlight the synergistic effects of pH pretreatment, chitosan flocculation, and green light irradiation in enhancing the BR of *E. gracilis*. The interactive effects between pH and chitosan concentration—as well as pH and green light irradiation time—were significant, indicating the importance of optimizing these factors simultaneously to achieve efficient harvesting. The synergistic integration of mildly acidic pH conditions, moderate chitosan concentrations, and prolonged green light exposure leveraged the unique properties of *E. gracilis* cells, resulting in superior harvesting performance compared to conventional methods.

### 3.3. Validation of the Optimized Conditions and Synergistic Mechanisms

The optimum conditions for the maximum BR were determined by numerical optimization using Design-Expert 13 software. The optimal levels of pH, chitosan concentration, and green light irradiation time were found to be 6.49, 46.10 mg·L^−1^, and 60 min, respectively. To verify the accuracy of the predicted values from the regression model, a verification experiment was conducted using the optimized parameters for *E. gracilis* biomass harvesting (in triplicate). The fit between the response surface methodology optimization and the experimental data was good, with an average BR of 93.07% for the three verification experiments, while the predicted maximum BR was 92.21%. To rigorously validate the predictive accuracy of our response surface methodology model, a paired *t*-test was performed comparing the predicted and experimental BR. The *p*-value exceeding the significance threshold of 0.05 indicated no statistically significant difference between the predicted and experimental values. This confirms the model’s high reliability and predictive power for estimating BR efficiency under the optimized harvesting conditions. The close alignment between predicted and experimental results further validates the robustness of the response surface methodology approach used in this study.

The high efficiency of this harvesting method can be attributed to the synergistic effects of pH adjustment, chitosan flocculation, and phototactic behavior. Each component plays a crucial role in the process, and their combined action results in a more effective harvesting strategy than any single mechanism alone. The optimum pH of 6.49 likely alters the surface charge of *E. gracilis* cells. At this pH, the cells may have a slightly negative charge, enhancing their interaction with the positively charged chitosan molecules [14]. This charge optimization sets the stage for effective flocculation without compromising cell viability. Chitosan, being a cationic polymer, interacts with the negatively charged cell surfaces, forming bridges between cells and promoting flocculation [12]. The flexible pellicle of *E. gracilis*, unlike rigid cell walls in other microalgae, may allow for more intimate contact between chitosan and the cell surface, enhancing flocculation efficiency [16]. The green light irradiation induces positive phototaxis in *E. gracilis*. While chitosan initiates flocculation via charge neutralization and bridging, the stirring step after chitosan addition ensures temporary cell suspension, allowing phototactic movement to persist during the early stages of flocculation. This directed movement increases cell–cell collisions and cell–chitosan interactions, promoting floc formation and growth before complete immobilization by the polymer matrix [9]. The light-induced movement may also help in breaking any weak flocs, allowing for the formation of larger, more stable flocs over time.

The synergy arises from the following mechanisms reinforcing each other: (1) pH adjustment optimizes the cell surface charge for chitosan interaction; (2) chitosan initiates floc formation; (3) phototaxis increases cell movement and collision frequency, enhancing floc growth and settlement. This combined effect explains the high harvesting efficiency achieved (93.07%) compared to methods relying on single mechanisms. Notably, chitosan addition precedes green light irradiation but the stirring step ensures the temporary suspension of cells, permitting phototactic movement during irradiation. This timing allows chitosan to initiate flocculation while phototaxis further concentrates cells into aggregates, overcoming potential mobility limitations caused by chitosan alone. The optimal pH likely strikes a balance between enhancing chitosan–cell interactions and maintaining cell motility for phototaxis. Too low a pH could neutralize cell surface charges, reducing chitosan binding, while too high a pH might affect cell viability or motility, hampering the phototactic response.

### 3.4. Photosynthetic Viability of Harvested Cells

**Figure 3:**
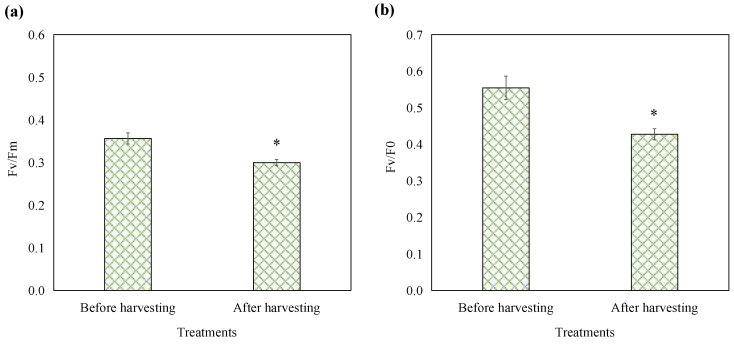
Effects of optimized harvest processing on the parameters Fv/Fm (**a**) and Fv/F0 (**b**) of *E. gracilis*. The asterisk denotes a significant difference compared to the pre-processing stage. Error bars represent the standard deviation (n = 3).

To assess the impact of the optimized harvesting treatment on the photosynthetic capabilities of *E. gracilis*, chlorophyll fluorescence parameters were measured before and after treatment. Fv/Fm represents the maximum photochemical quantum yield of PSII, reflecting the intrinsic photon conversion efficiency of the PSII reaction center, also known as the primary photochemical conversion efficiency of PSII. Fv/F0 represents the potential activity of PSII [17]. As shown in Figure 3, prior to harvesting, the Fv/Fm value of *E. gracilis* was 0.36, which falls within the range of photosynthetic efficiency observed in the existing literature. Recent studies have reported Fv/Fm values of healthy *E. gracilis* varying from 0.3 to 0.64 [18,19], a variability attributable to differences in experimental conditions, strain characteristics, and environmental factors. The harvesting treatment resulted in a decline of 15.90% in Fv/Fm and 22.91% in Fv/F0. Despite these changes, the *E. gracilis* cells maintained substantial photosynthetic activity. This observation is critical for evaluating the biocompatibility and potential cytotoxicity of the harvesting method. Conventional harvesting techniques such as centrifugation, filtration, and aluminum sulfate salts addition can cause cell damage and some chemical flocculants are even toxic and harmful, rendering the biomass unsuitable for reuse in cultures or applications in the food industry [2]. In contrast, this mild harvesting approach leveraging *E. gracilis’* natural phototaxis and pH preconditioning in combination with the biocompatible chitosan flocculant allowed the harvested cells to maintain certain levels of photosynthetic activity. This biocompatible nature is a significant advantage over conventional harvesting techniques that can compromise cell viability and limit the range of microalgal applications.

By maintaining a certain level of photosynthetic activity post-harvesting, the recovered *E. gracilis* biomass remains a viable candidate for various high-value applications in the food, feed, and nutraceutical sectors, which demand minimal chemical treatments and the preservation of cellular integrity [7]. The mild nature of the phototaxis–pH–flocculation treatment enables the effective recovery of microalgal biomass while retaining its nutritional and biochemical properties, thereby broadening its potential commercial applications.

### 3.5. Economic Analysis

The cost of the flocculant is an important consideration as the biomass recovery stage may account for up to 60% of the total production cost [2]. The high cost of flocculants is economically unfeasible and, in turn, limits their applicability in full-scale processes. Therefore, an economic analysis was performed to evaluate the cost-effectiveness of the optimized harvesting method. The primary cost components considered were the chitosan biopolymer pH adjustment reagents and the energy consumption associated with green light irradiation. Based on the optimized chitosan concentration of 46.10 mg·L^−1^ and the assumed commercial chitosan cost of 20 USD/kg [8], the chitosan cost per kg of harvested biomass was calculated to be USD 1.87. Excluding other factors such as heat dissipation, drive circuits, etc., considering an energy consumption rate of 0.2 kWh/m^3^ for green light irradiation and an electricity cost of 0.10 USD/kWh, the energy cost per kg of harvested biomass was estimated to be USD 0.43. The cost of pH pretreatment consumed for recovering 1 kg of *E. gracilis* dry biomass was calculated to be approximately USD 0.05. Thus, the total harvesting cost per kg of dry biomass was approximately USD 2.35. Currently, there have been no reports on a cost analysis of harvesting *E. gracilis*. Research on the harvesting of *E. gracilis* is limited and mostly relies on the outdated literature. This study fills the gap in this area. In comparison to the cost of harvesting other economically important algae using chitosan alone, the optimized method in this study results in lower costs. For instance, Gupta et al. [20] found that the cost of harvesting 1 kg of *Scenedesmus* sp. biomass using chitosan was USD 51.02, which is much higher than the cost in this study. It is important to note that the cost analysis presented here is based on certain assumptions and may vary depending on factors such as the scale of operation, location, and availability of resources. However, the overall trend suggests that the optimized harvesting method, integrating chitosan flocculation and green light irradiation, offers a cost-effective solution compared to traditional approaches. The economic viability of this method is further supported by the use of biodegradable and non-toxic chitosan as the flocculant, eliminating the need for costly chemical treatments and minimizing the environmental impact. Additionally, the utilization of green light irradiation leverages the unique phototactic behavior of *E. gracilis*, reducing the energy consumption associated with conventional harvesting techniques. Utilizing natural light or green light within natural light to drive the autonomous harvesting of *E. gracilis* could significantly enhance cost-effectiveness and prove advantageous for large-scale outdoor harvesting operations.

### 3.6. Scalability Considerations

While the experiments were conducted at the laboratory scale, the potential for scaling up this harvesting method warrants careful consideration. The three main components—pH adjustment, chitosan addition, and light irradiation—each present unique challenges and opportunities for scale-up. A pH adjustment can be relatively straightforward to scale, requiring larger volumes of acid or base and appropriate mixing systems, though maintaining a uniform pH throughout larger volumes may necessitate a careful design of the mixing protocols. Chitosan addition can be scaled by increasing the volume of chitosan solution proportionally but ensuring a uniform distribution in larger volumes may require an optimization of the mixing strategies, potentially including the use of static mixers or multi-point injection systems [21]. The most challenging aspect for scale-up is likely the light irradiation component. In larger volumes, light penetration becomes a limiting factor due to self-shading effects, which could potentially be addressed through several strategies: (1) the use of multiple light sources or LED arrays surrounding the cultivation vessel; (2) the implementation of internal light guides or optical fibers to distribute light more evenly; (3) the design of shallow, large surface area reactors to maximize light exposure; or (4) the development of sequential harvesting strategies where smaller volumes are exposed to light in stages [22,23]. Additionally, the energy consumption for large-scale light irradiation needs to be carefully evaluated to ensure the process remains economically viable. The interaction between these scaled-up components may also introduce new dynamics that could affect harvesting efficiency, such as changes in floc formation and settling rates in larger volumes. Therefore, pilot-scale studies will be crucial to optimize the process parameters for industrial-scale applications, focusing on maintaining high harvesting efficiency while minimizing energy input and operational costs. Further studies should also address practical considerations such as the design of large-scale mixing and light distribution systems as well as the development of automated control systems to maintain optimal conditions throughout the scaled-up process.

### 3.7. Comparative Analysis with Other Harvesting Methods

The proposed harvesting method, synergistically integrating pH pretreatment, chitosan flocculation, and green light irradiation, exhibits several notable advantages over conventional and emerging techniques employed for microalgal BR. A comprehensive comparison with other harvesting approaches, summarized in Table 3, highlights the merits and distinctive features of this method.

In terms of BBR, the optimized conditions (pH 6.49, 46.10 mg·L^−1^ chitosan, 60 min green light) achieved an impressive 93.07% recovery for *E. gracilis* biomass. This performance is comparable or superior to many established methods, including bioflocculation of *E. gracilis* by fungal filaments (62–75%) [24]; phototaxis alone for *E. gracilis* harvesting (70%) [9]; organic flocculation with chitosan for other species like *C. vulgaris* (99.1%) [12]; and magnetic flocculation for *Chlorella pyrenoidosa* (97%) [25]. However, techniques like biosorption [26] and bioflocculation [27,28] have demonstrated marginally higher efficiencies for certain microalgal species, while their applicability to *E. gracilis* still lacks relevant evidence. Notably, chitosan-only flocculation studies report the BR ranging from 85% to 99% depending on species and conditions [12,14,29]. Our integrated approach achieves comparable efficiency (93.07%) while reducing chitosan dependency by ~50% compared to typical doses (e.g., 80–100 mg·L^−1^ [8,20]). This demonstrates the advantage of combining chitosan with pH adjustment and phototaxis to enhance cost-effectiveness and sustainability.

A notable advantage of the proposed method is its relatively short harvesting time of 1 h, significantly faster than approaches like bioflocculation (1–3 h) [24], phototaxis (24 h) [9], and bacteria–fungi co-flocculation (24 h) [30]. This duration is comparable to pH-induced flocculation (1 h) [31] and some organic flocculation methods [32,33] but longer than centrifugation (2–5 min) [29], magnetic flocculation (2 min) [34], and electrolytic flocculation (20 min) [35].

From an environmental perspective, the proposed method stands out by utilizing the biodegradable and non-toxic biopolymer chitosan as the primary flocculant, in contrast to inorganic flocculants or coagulants like aluminum or iron salts, which can be toxic and generate harmful sludge [36]. Additionally, the incorporation of green light irradiation contributes to the method’s sustainability and energy efficiency. A unique feature of this approach is the synergistic combination of pH pretreatment, chitosan flocculation, and leveraging *E. gracilis’* phototactic behavior through green light irradiation. This innovative strategy, tailored specifically for *E. gracilis*—a microalga lacking a rigid cell wall—distinguishes it from conventional techniques and demonstrates the potential for adapting the principles of pH pretreatment, organic flocculation, and phototaxis to other phototactic microalgae species.

In summary, the proposed harvesting method exhibits a unique synergistic approach, achieving high efficiency, a short processing time, environmental friendliness, and potential versatility for application to other phototactic microalgae species. While it may not outperform certain methods in specific aspects like harvesting time or efficiency for different species, its combination of advantages establishes it as a promising solution for the efficient and sustainable recovery of *E. gracilis* biomass.

**Table 3:** Comparison of the proposed harvesting method with other harvesting strategies for different microalgal species.

Methods	Principles	Microalgal Species	BBR	Harvesting Time	Refs.
pH pretreatment combined with chitosan flocculation and green light irradiation: pH 6.49; 46.10 mg·L^−1^ chitosan; 60 min green light	Phototaxis and organic flocculation (cationic)	*E. gracilis*	93.07%	1 h	This study
*Ganoderma lucidum*, *Pleurotus ostreatus*, and *Penicillium restrictum* flocculate *E. gracilis*	Bioflocculation	*E. gracilis*	62–75%	1–3 h	[24]
24 h green light irradiation	Phototaxis	*E. gracilis*	70%	24 h	[9]
pH 5; 10 mg·L^−1^ chitosan; 45 min flocculation	Organic flocculation (cationic)	*C. vulgaris*	99.1%	0.75 h	[12]
Cationic organic polymer γ-PGA induced the neutralization of the surface charge	Organic flocculation (cationic)	*C. vulgaris*	>90%	2 h	[32]
Encapsulation of microalgae by nanofibrous structure formation of cellulose nanofiber	Organic flocculation (non-cationic)	*C. reinhardtii*	Good (no data)	2 h	[33]
pH 9; 0.738 g·L^−1^ magnetic flocculant nanoparticles; 0.5 T magnetic field intensity	Magnetic flocculation	*C. pyrenoidosa*	97%	/	[25]
pH 7; 150 mesh size; and 10 mg·L^−1^ methyl-eggshell membrane	Biosorption	*C. vulgaris*	99%	10 min	[26]
pH 2; chitosan and ferric chloride dual-modified biochar	Magnetic flocculation	*C. pyrenoidosa*	96.9%	2 min	[34]
pH 7; bacteria–microalgae ratio of 1.6:1; microalgae–fungi ratio of 333:1; glucose concentration of 1.47 g·L^−1^	Bacteria–fungi co-flocculation	*C. pyrenoidosa*	97.45%	24 h	[29]
Aluminum-based coagulant (AlCl_3_) neutralized and reduced the surface charge	Inorganic flocculation (cationic)	*Chlorella minutissima*	>90%	1–4 h	[36]
Centrifugation at 500–1000× *g*	Centrifugation	Most algal species	80–90%	2–5 min	[29]
*Bacillus licheniformis* CGMCC 2876 produced bioflocculant γ-PGA	Bioflocculation	*Desmodesmus* sp.	98.20%	1–2 min	[28]
Aggregation of negatively charged microalgae and positively charged fungi Aspergillus oryzae UMN F07	Bioflocculation	*C. vulgaris* UMN235	99.2% for heterotrophy and 63% for autotrophy	2–4 d	[27]
Movement of microalgae to the anode to neutralize the carried charge and form aggregates	Electrolytic flocculation with aluminum electrodes	*Scenedesmus* sp.	98.5%	20 min	[35]

## 4. Conclusions

This study presents an innovative approach for the efficient harvesting of *E. gracilis* biomass, uniquely combining charge neutralization, biopolymer flocculation, and phototaxis-induced cell aggregation. The synergistic effects of these mechanisms contribute to the superior harvesting performance, achieving a high BR while maintaining cell integrity. This novel method addresses the specific characteristics of *E. gracilis*, such as its flexible pellicle and phototactic behavior, offering a tailored solution for this important microalgal species.

The biocompatibility of our approach is confirmed through chlorophyll fluorescence analysis, demonstrating that harvested cells retain their photosynthetic capability. This preservation of cell viability is crucial for various applications of *E. gracilis* biomass, particularly in the production of high-value compounds and nutraceuticals. Despite being conducted at the laboratory scale, the method shows promise in terms of cost-effectiveness, with a low estimated harvesting cost of 2.35 USD per kg of dry biomass.

Furthermore, the principles underlying this method hold potential for application to other phototactic microalgae species lacking rigid cell walls. The combination of charge-based flocculation and light-induced movement could be adapted to various photosynthetic microorganisms, opening new avenues for efficient and gentle harvesting across a range of microalgal species. This adaptability, coupled with the method’s economic advantages and environmentally friendly nature, positions it as a sustainable strategy for advancing microalgal biotechnology and contributing to the development of bio-based products.

## Figures and Tables

**Table 1:** Box–Behnken central composite experimental design.

No.	A	B	C	BR/%
1	5	20	35	77.14
2	9	20	35	49.85
3	5	80	35	60.85
4	9	80	35	71.75
5	5	50	10	64.74
6	9	50	10	62.74
7	5	50	60	82.23
8	9	50	60	64.00
9	7	20	10	78.10
10	7	80	10	82.07
11	7	20	60	83.15
12	7	80	60	85.12
13	7	50	35	92.22
14	7	50	35	88.31
15	7	50	35	91.32
16	7	50	35	85.90
17	7	50	35	92.20

**Table 2:** ANOVA for the quadratic model.

Source	Sum of Squares	df	Mean Square	F-Value	*p*-Value
Model	2533.45	9	281.49	41.74	<0.0001
A: pH	167.63	1	167.63	24.85	0.0016
B: chitosan concentration	16.68	1	16.68	2.47	0.1599
C: green light irradiation time	90.12	1	90.12	13.36	0.0081
AB	364.62	1	364.62	54.06	0.0002
AC	65.85	1	65.85	9.76	0.0167
BC	1.0000	1	1.0000	0.1483	0.7116
A^2^	1582.63	1	1582.63	234.66	<0.0001
B^2^	137.04	1	137.04	20.32	0.0028
C^2^	19.92	1	19.92	2.95	0.1294
Residual	47.21	7	6.74		
Lack of fit	16.03	3	5.34	0.6858	0.6060
Pure error	31.18	4	7.79		
Cor total	2580.66	16			

## Data Availability

Data will be made available on request.
